# Sequential occurrence of recurrent Sweet syndrome and erythema nodosum without an underlying secondary cause: a case report

**DOI:** 10.1186/s13256-022-03282-1

**Published:** 2022-02-25

**Authors:** Chamila Mettananda, Hansika Peiris, Ahamed Uwyse

**Affiliations:** 1 Department of Pharmacology, Faculty of Medicine, University of Kelaniya, Ragama, Sri Lanka; 2 North Colombo Teaching Hospital, Ragama, Sri Lanka

**Keywords:** Fever, Rash, Sweet syndrome, Erythema nodosum red-eye, Case report

## Abstract

**Background:**

Sweet syndrome is a rare cause of acute fever and painful erythematous skin plaques. Erythema nodosum is acute or chronic tender erythematous skin nodules of bilateral shins. The concurrent presence of both dermatoses is rare but reported in the literature. There are no reported cases of recurrent and sequential Sweet syndrome and erythema nodosum without an underlying secondary cause.

**Case presentation:**

We report the case of a 64-year-old Asian woman, who had possible Sweet syndrome 12 years ago and biopsy-proven erythema nodosum 5 years ago, presenting with an acute episode of Sweet syndrome. Extensive investigations did not reveal any underlying secondary cause.

**Conclusions:**

Recurrent Sweet syndrome and sequential presence with erythema nodosum raises suspicion if Sweet syndrome and erythema nodosum are different presentations of one disease, which warrants further study. This case proves that recurrent Sweet syndrome and erythema nodosum can occur in healthy individuals without an underlying malignancy or secondary cause.

## Background

Sweet syndrome (SS) is an acute febrile neutrophilic dermatosis. It is a rare differential diagnosis in a patient with fever, usually presenting as fever and acute painful erythematous skin plaques, arthralgia, and episcleritis [1, 2]. SS presenting following infections or an immunological cause is called classic Sweet syndrome. It can also be malignancy induced [3, 4] or drug induced [2]. Pathologically, there is edema on the upper dermis and intense neutrophilic infiltration [2]. Recurrent Sweet syndrome is reported in about one-third of patients [2].

Erythema nodosum (EN) is acute or chronic tender erythematous skin nodules of bilateral shins [5]. It has been shown to follow streptococcal pharyngitis, but may precede tuberculosis, bacterial or deep fungal infection, sarcoidosis, inflammatory bowel disease, or cancer; however, it is mainly idiopathic. Pathologically, it shows granulomatous panniculitis [6, 7].

The concurrence of both dermatoses is rare but reported in the literature: eight cases without underlying malignancies [8], one case following sarcoidosis [9], one case following Crohn’s disease [10], two cases following streptococcal upper respiratory infections [9, 11], and one case following acute myeloid leukemia [12]. Both are reactive dermatoses, but the pathogenesis is not well understood [13–15].

Sequential presence of SS and then EN is even rarer; there is one case reported in a patient with myeloid leukemia [16]. Our case is the first to report sequential presentation of SS and EN without an underlying malignancy or secondary cause.

## Case presentation

**Fig. 1:**
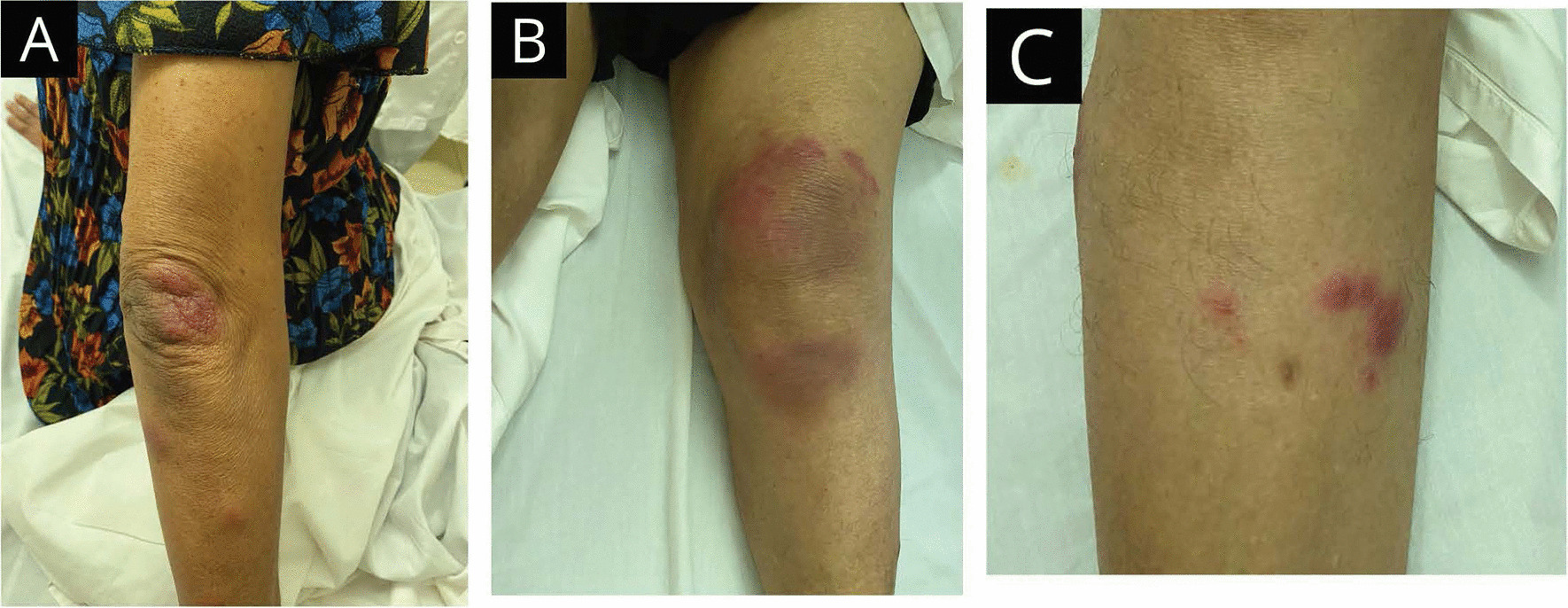
At presentation—4 days after onset of fever and rash. Tender erythematous skin plaques over (**A**) right elbow joint, (**B**) left knee joint, and (**C**) right shin

**Fig. 2:**
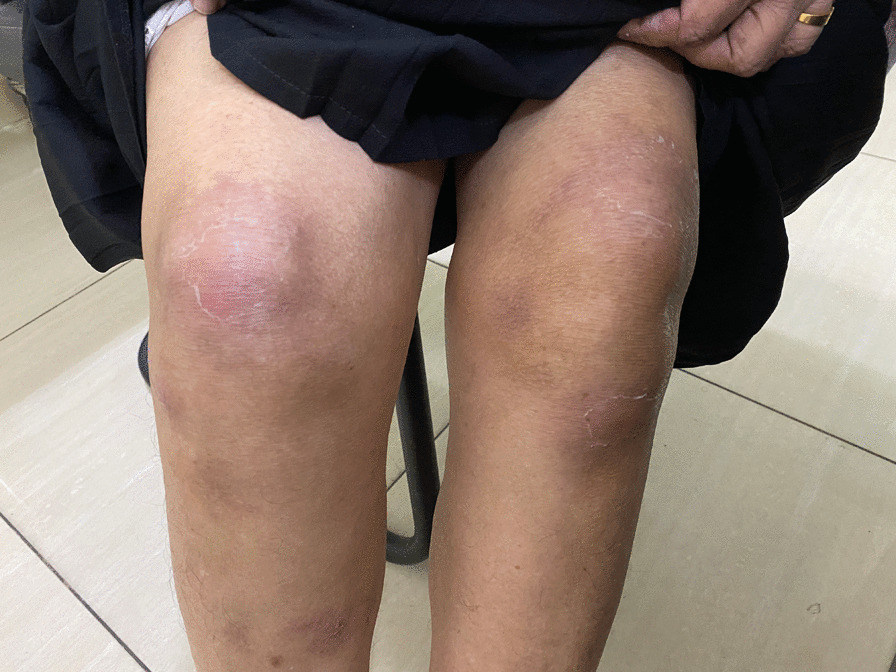
After treatment—14 days after the onset of illness. Healing skin rash without scarring

A 64-year old, previously healthy, Asian woman presented with a 4-day history of fever, and multiple, between 1 and 3 cm in width, irregularly shaped, tender, red-color, palpable, non-scaly skin plaques over dorsal aspects of knees, elbows, and shins with ankle joint pain and left-sided painless red eye (Fig. 1). There was no clinical proximal muscle weakness or racoon eyes. The rest of the examination was normal. Initially, an infectious etiology was suspected. She had high inflammatory markers, erythrocyte sedimentation rate (ESR) 117 mm after 1 hour, C-reactive protein (CRP) 168 mg/dl, white blood cells 12.5 × 10^3^/µL, neutrophils 67%, but a negative septic screen, and creatine phosphokinase (CPK) 72 U/L. She was promptly started on intravenous co-amoxiclav and oral ciprofloxacin to cover an infection. On further questioning, she had had a fever with a similar skin rash, involving upper arms and legs, treated in hospital for about a week 12 years ago for which the records were not available. However, she was treated for biopsy-proven erythema nodosum 5 years ago, which settled without any consequences. With this past medical history, Sweet syndrome was suspected, and she was started on oral methylprednisolone 16 mg daily while being on antibiotics. The symptoms improved dramatically in 2 days, and skin biopsy was not performed as the patient refused it with quick symptom resolution. However, diagnostic criteria were fulfilled and were pointing to a diagnosis of Sweet syndrome. The plaques disappeared completely, leaving only skin discoloration by 1 week (Fig. 2). Inflammatory markers also improved significantly by 1 week; ESR 60 mm after 1 hour and CRP 8 mg/dl. The patient was treated with methylprednisolone 16 mg daily for 2 weeks, and it was tailed off over 6 weeks. A comprehensive set of investigations including liver biochemistry, serum amylase, blood picture, chest X-ray, and ultrasound (US) abdomen did not find any underlying secondary cause for the recurrent SS and EN in this patient. The patient was followed up 9 months after the current presentation, and she was asymptomatic with an ESR of 20 mm after 1 hour.

## Discussion

This patient had classic symptoms of Sweet syndrome involving skin, eyes, and joints. Laboratory investigations were supportive of the diagnosis and excluded other differential diagnoses. Swift response of the disease to steroids helped the diagnosis. She did not have any evidence of underlying malignancy, nor was she on any medication known to precipitate Sweet syndrome. Therefore, classic Sweet syndrome was diagnosed on criteria [17]. We did not find any precipitant for classic SS such as respiratory infection, or other inflammatory diseases. She had EN 5 years ago. The first presentation 12 years ago was also likely to be SS but no skin biopsy had been done to confirm.

The concurrent presence of SS and EN is reported in the literature [8–12]. Sequential presence of SS and EN is reported in a patient with myeloid leukemia [16]. This is the first case reported of sequential presentation of SS and EN in an otherwise healthy individual without any underlying secondary cause, observed over 12 years duration.

The possible recurrent nature of SS and sequential presence of EN need further investigation into the pathogenesis of the disease to rule out if this is a spectrum of one disease and not two clinical entities. SS and EN have several features in common: both are reactive dermatoses; the usual stimuli are the same; skin manifestations are very similar with the only difference being in the distribution of the skin lesions, with EN usually being confined to the dorsal aspect of lower limbs; and both respond to the same treatment, with steroids, either oral or topical, being useful in the management of these skin disorders [18, 19]. We considered our patient at the current presentation as having concurrent SS and EN, yet a misdiagnosis is possible, as she may have had SS only, with some features of EN. However, the suspicion for it being a spectrum of one same disease is even higher when SS and EN present sequentially, which does not give room for misdiagnosis EN. In SS, there is a dense, mature, neutrophilic infiltration of the upper dermis [2, 20, 21]. EN shows a neutrophilic infiltrate in the early stage, and though histological presentation can vary, its hallmark is granulomatous panniculitis called Miescher’s radial granulomas [5, 6]. EN is reported to show lobular neutrophilic panniculitis with suppuration, small-vessel vasculitis, and even medium-vessel arteritis, although rarely [22, 23].

A minority of cases with SS and EN can be associated with malignancies. Around 16% of Sweet syndrome cases are malignancy related and can precede, follow, or appear concurrent with the diagnosis of the patient’s neoplasm, especially hematological malignancies [4]. Many cases of EN are idiopathic and less associated with malignancies compared with SS [5, 6]. EN can be a cutaneous marker of lymphoma/leukemia [24, 25] and carcinoid/colorectal/pancreatic cancers [26, 27] and can be a marker of recurrence of those malignancies. However, the presence of recurrent SS and EN in the same patient without an underlying secondary cause, as we reported, suggests that patients may have recurrent SS or concurrent/sequential SS and EN that is not always indicative of an underlying chronic disease or a malignancy; instead, it could be idiopathic.

## Conclusions

There are no reported cases of recurrent and sequential SS and EN without an underlying secondary cause. We report a 64-year-old, otherwise healthy, Asian woman with sequential recurrent SS and EN over 12 years without an underlying secondary cause. Recurrent SS and sequential presence with EN raise the suspicion of SS and EN being different presentations of one disease, which warrants further study. This case proves that recurrent SS and sequential SS and EN may occur in healthy individuals without an underlying malignancy or secondary cause.

## Data Availability

Not applicable.
